# Comparative Analysis of the Application of Five-, Seven- and Nine-Roll Sheet Straightening Using Numerical Tools

**DOI:** 10.3390/ma19061053

**Published:** 2026-03-10

**Authors:** Grzegorz Stradomski, Sebastian Mróz, Piotr Szota, Tomasz Garstka, Jakub Gróbarczyk, Radosław Gryczkowski

**Affiliations:** 1 Faculty of Production Engineering and Materials Technology, Czestochowa University of Technology, 19 Armii Krajowej Av., 42-201 Czestochowa, Poland; sebastian.mroz@pcz.pl (S.M.); piotr.szota@pcz.pl (P.S.); tomasz.garstka@pcz.pl (T.G.); 2 Serwistal Sp. z. o.o., 2A Dojazdowa Str., 19-300 Ełk, Poland; j.grobarczyk@serwistal.pl (J.G.); r.gryczkowski@serwistal.pl (R.G.)

**Keywords:** sheet straightening, numerical analysis, steels, stresses, geometric surface defects

## Abstract

The paper presents the results of a numerical analysis of the roller straightening process. The study evaluated straightening systems consisting of 5, 7, and 9 rollers. The assessment was based on real data obtained under industrial conditions. The research involved the use of two widely used structural steel grades, namely S235JR and S500MC. As part of the study, an analysis of the anisotropy of sheet properties within the coil volume was also conducted. Additionally, the surface topography of the sheets was analyzed and subsequently used in the numerical simulations. The inclusion of real data regarding both material anisotropy and actual geometry allowed for an increase in calculation accuracy. Furthermore, investigations were carried out to analyze the method of implementing material data into the mathematical model. The work also analyzed the accuracy of the obtained numerical results based on material data from the traditional method of introducing material information in the form of an approximated curve. Numerical studies confirmed by a physical test developed by the authors, which can be easily implemented in industrial conditions, confirmed the research assumptions and literature data. The main advantage of the presented solutions is their relative ease of implementation and use in manufacturing facilities with limited research facilities.

## 1. Introduction

The production of sheet metal in the form of flat sheets, used as feedstock for processes such as laser cutting or press forming, requires several technological stages resulting from the fact that the material is delivered in coils. Sheet metal coils offer undeniable advantages, such as easier transportation. However, after uncoiling, the sheets exhibit waviness and non-uniform residual stress levels both across their cross-section and along their length. This is, among other things, a remnant of the rolling and coiling process, commonly referred to as the “coil memory,” which contributes to the formation of geometric defects and increased, uneven distributions of residual stresses [1,2,3].

Producing high-quality sheets requires identifying the defects that occur after uncoiling as well as after preliminary straightening in a roller straightener. An important aspect is the evaluation of sheet flatness after uncoiling, before preliminary straightening in roller straighteners, since at this stage it is crucial to eliminate a significant portion of geometric defects and reduce the level of residual stresses, which will subsequently affect the residual stress state during tension straightening [4,5,6].

**Figure 1:**
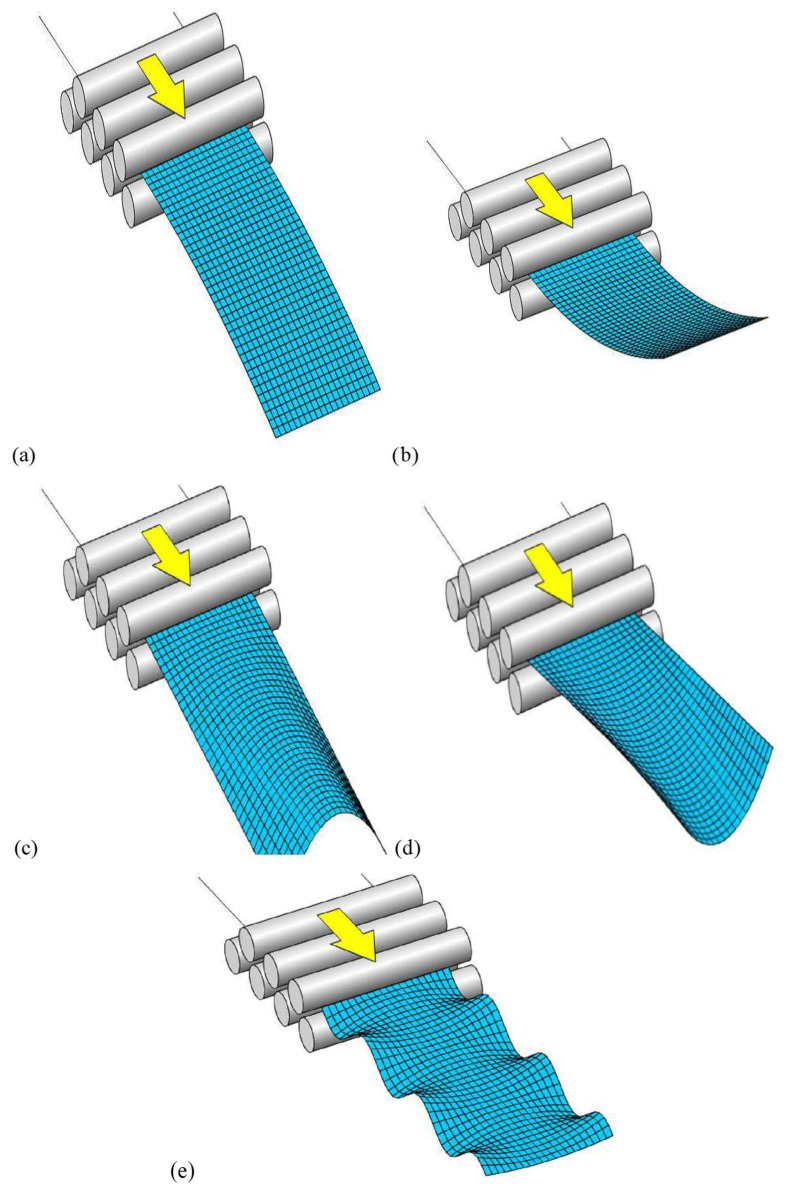
Schematic view of defects: (**a**,**b**) longitudinal defects in sheets—(**a**) downward bending direction—positive, (**b**) upward bending direction—negative; (**c**,**d**) transverse defects in sheets in the form of bending—(**c**) downward bending direction—positive, (**d**) upward bending direction—negative; (**e**) edge waviness in sheets. Own elaboration based on [7].

Implementing any improvements in the straightening process requires determining the initial geometry of the sheet. The most characteristic defects found in cold-rolled and hot-rolled sheets include longitudinal bowing, transverse bowing, edge waviness, and center waviness. A schematic view of these defects is shown in Figure 1.

The characteristics of the technological process of producing flat products, particularly in the finishing rolling stage, are closely related to the non-uniform deformation occurring in the roll gap. At the exit of the rolling mill, the transverse contour of the strip is uneven, which is the main cause of residual stresses along its length. In the case of a convex contour, compressive stresses occur at the edges of the sheet, while tensile stresses develop in the center. If these stresses exceed the critical buckling strength of the strip, edge deformation may occur in the form of waviness or cyclic bulging in the central part of the strip, resulting from partial stress relaxation.

An additional source of stress during rolling is deformation caused by coiling and uncoiling of the strip, as well as by uneven cooling of the coiled sheet during hot rolling. As a result of all these technological processes [4,5,8], a complex residual stress state exists in the sheet strip, leading to shape deformations that must be removed to ensure the semi-finished product meets quality requirements [9,10].

Many authors [11,12,13] have studied the possibilities of using roller systems to reduce residual stresses. For particular note is the study [11], in which the authors used numerical tools to model straightening in multi-roller systems. This is important because such systems offer great flexibility for eliminating many types of defects. The defects shown in Figure 1 can be significantly reduced or even completely eliminated [14,15,16] through roller straightening, especially when combined with tension straightening. However, it is necessary to identify the type and magnitude of defects after uncoiling in order to choose the appropriate straightening methodology (in the case of a preliminary roller straightener—to determine the correct roller positions).

In addition to visible geometric defects, sheets also exhibit “hidden defects” in the form of residual stresses [17,18,19], which, when too high, can cause deformation—particularly during cutting. Such defects can be eliminated during the tensioning process. Their origin lies in non-uniform rolling conditions, and in the case of hot-rolled sheets, additional factors such as temperature non-uniformity further contribute to their formation. Uneven temperature distribution in the sheet leads to residual stresses, which often affect sheet geometry as well.

However, in order to apply the appropriate straightening method [20,21,22] (in the case of a roller straightener—proper roller positioning), it is necessary to determine the type and magnitude of defects after uncoiling.

Excellent results in process design, acceleration, and cost reduction in research can be achieved through the use of numerical tools, such as the finite element method (FEM). Many authors, including the authors of this paper, have used FEM-based tools to solve similar problems [23,24,25].

It is evident that internal stresses, which in the described case are a primary source of geometric defects, result from manufacturing processes. Many authors, in their works regarding stresses across various product groups, have described both their effects on properties or microstructure [26,27,28] and on defects, such as surface irregularities [29,30,31]. Another element to be considered in the field of internal stresses is their origin resulting from heat or thermomechanical treatment [32,33,34]. The observed effects—manifesting as changes in properties, microstructural non-homogeneity, delamination, and cracking—are unfavorable.

In industrial conditions, particularly on production lines dedicated to formatting sheet metal into sheets for further production, it is impossible to fully investigate stress distribution directions or determine their root causes. Therefore, it is crucial to develop solutions that allow for their reduction in a relatively universal manner. In this study, the authors focused on the analysis of such a production line; consequently, the origin of the stresses, regardless of its nature, was not the primary evaluation criterion. Among the most commonly used industrial techniques for reducing internal stress states are technologies utilizing multi-roller systems. The research presented in this paper is part of a much larger project, the results of which led to significant positive outcomes, namely the reduction in geometric defects in metal sheets. The multi-roller system itself, while critical, was only one component of this solution, which constitutes the intellectual property of Serwistal and is legally protected; therefore, it is not possible to present all elements of the production line.

## 2. Research Material and Preliminary Tests

The material used for the study consisted of sheets uncoiled from coils made of structural steels S235JR + AR and S500MC. The selection of research materials was driven by the fact that these two steel grades account for a significant share of Serwistal’s current production. Another important consideration was that both materials were subjected to similar finishing treatments. Both steels were in the hot-rolled condition and can be subjected to an annealing process after rolling; however, to reduce production costs, they are typically rolled into coils without additional heat treatment. During hot rolling, the rolls are cooled with water, and in most cases, the edges of the sheet cool faster than the central part, which may lead to differences in properties between the central and edge regions. Neither the microstructure nor the material texture was analyzed within this work or in the project (POIR.01.01.01-00-0477/18). Such analyses are not feasible under industrial conditions; furthermore, the material manufacturer is responsible for both the quality of the microstructure and the chemical composition. This work presents results that can be obtained quite quickly and easily in industrial conditions. The chemical composition of the tested steels, determined using spark spectroscopy, is presented in Table 1.

As part of the first stage of the research, the anisotropy of the material properties was evaluated. The tests were carried out using Vickers hardness measurements with a Matsuzawa Via S hardness tester (Matsuzawa, Akita, Japan) and a static tensile test using an MTS E45.305 testing machine (MTS Systems, Eden Prairie, MN, USA).

Hardness test samples were taken from six areas of the coil—the beginning (b), middle (m), and end (e). Assuming symmetry of properties across the sheet width, the number of samples in the transverse direction was limited to two—one taken from the edge and one from the center of the strip. The areas from which hardness samples were taken covered the same zones of the beginning, middle and end of the coil that were examined later in the article. These locations were selected because the literature data, observations from sheet metal manufacturer Serwistal (Ełk, Poland), and preliminary studies indicated that these areas differ significantly in surface geometry. Additionally, as also presented in works [4,5], it was determined that these places are characterized by significant unevenness in internal stresses. Table 2 presents the sample identification and the corresponding area of the sheet (edge or center) from which each specimen was taken, while Table 3 and Table 4 show the measured results obtained.

The obtained data indicate that the greatest differences in values were recorded at the beginning and end areas of the coil. These differences were observed regardless of the steel grade, which is closely related to the rolling process technology. Hot-rolled sheets exhibited greater hardness non-uniformity across the strip width, with differences reaching up to 9.7%.

The analysis of the anisotropy of mechanical properties aimed not only to investigate the hypothesis regarding significant differences between individual areas of the ring, but also to determine the extent of these differences. Only this knowledge allowed for an assessment of the feasibility of numerical studies and the identification of necessary simplifications. This is particularly crucial because, while incorporating the actual geometry of an object into numerical tools is a relatively straightforward task, implementing significant anisotropy is essentially impossible or nearly so. The use of relatively simple tests, such as hardness and static tensile testing, served a dual purpose. Firstly, these tests are feasible under production conditions; secondly, they give a clear result. As will be explained further in this paper, the development of a tabular material model—as the most effective method for defining material anisotropy parameters—enabled its implementation in numerical research. Due to the fact that during the implementation of project POIR.01.01.01-00-0477/18, a sheet metal straightening system was designed and developed based on two components consisting of a preliminary multi-roller leveler and a final tension leveler, knowledge regarding the non-uniformity of mechanical properties in the longitudinal direction was essential. The multi-roller straightening system presented in this work served as a preliminary stress relief stage. Since this stage is important but not the primary factor, the research was simplified to focus solely on longitudinal properties. Consequently, it was possible to introduce a simplification by limiting the study to the investigation of properties in the longitudinal direction. By combining the roller system with the final tension stage, it is possible to orient the stresses and ultimately obtain high-quality products characterized by the absence of surface defects. Related studies [4,5] provide additional data on this project, specifically regarding stress states measured using the Barkhausen noise method.

**Figure 2:**
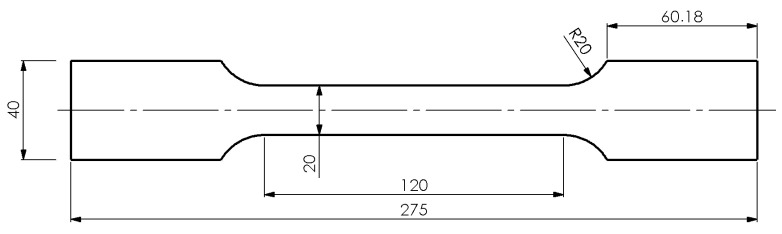
Dog-bone specimen used in the tests.

Subsequently, tensile tests were carried out to determine the actual anisotropy of mechanical properties (Ys—yield strength; UTS—ultimate tensile strength). The samples used for testing at room temperature are shown in Figure 2.

The sampling locations included the beginning, middle, and end of the coil—both from the central part of the sheet and near its side edges. Three samples were prepared from each location. The results are presented in Table 4 and Table 5.

The data presented in Table 4 and Table 5 show that the greatest differences in yield strength and tensile strength values—regardless of the steel grade—were observed across the sheet width at the beginning and end of the coil. This results from the rolling process technology. The differences across the width reached nearly 10%, while smaller variations, up to about 6%, were observed along the coil length.

For accurate numerical modeling, it was necessary to adopt a certain degree of data homogenization. To achieve this, the obtained values were averaged. Due to the significant number of samples intended for testing, dedicated computer software was developed to enable automatic numerical analysis of the collected data. The data, initially stored as text files, were processed into a structured table containing the essential parameters required to generate flow stress curves for each steel grade. Additionally, the results were averaged within each steel grade category.

**Figure 3:**
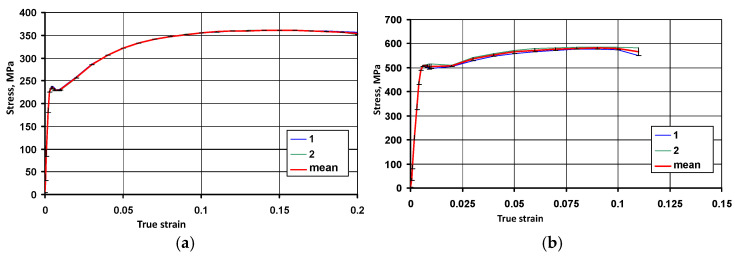
Averaged flow stress curves obtained from the results of the static tensile test: (**a**) S235JR + AR, (**b**) S500MC.

Figure 3 presents examples of flow stress curves obtained for the selected steel grades, while Table 6 shows their averaged characteristics.

For the obtained curves, an exponential function was fitted using the developed computer program, as it most accurately represented the experimental data. An analysis of different forms of exponential functions, varying in the number of coefficients, showed that the four-parameter exponential function provided the best fit to the actual curve behavior. The function takes the following form:

(1) $$\sigma_{f}=K\cdot \varepsilon^{m_{1}}\cdot {exp}^{\frac{m_{2}}{\varepsilon }}\cdot {exp}^{\varepsilon m_{3}},[\mathrm{MPa}]$$

The obtained flow stress curves were then subjected to numerical analysis using the developed software. A least-squares approximation was performed using a four-parameter quadratic exponential function dependent on strain, which made it possible to determine the coefficients of function (1): K, m_1_, m_2_, and m_3_. The applied method for determining the function coefficients minimizes the deviation error between the fitted function and the actual flow stress curves. Table 3 presents the coefficients of function (1) for the selected steel grades.

The developed mathematical model of flow stress enables computer simulations of sheet behavior using a deformable body model in an elasto-plastic state. The elastic state is represented by Young’s modulus, while the deformation range beyond the yield point is described by the flow stress function.

**Figure 4:**
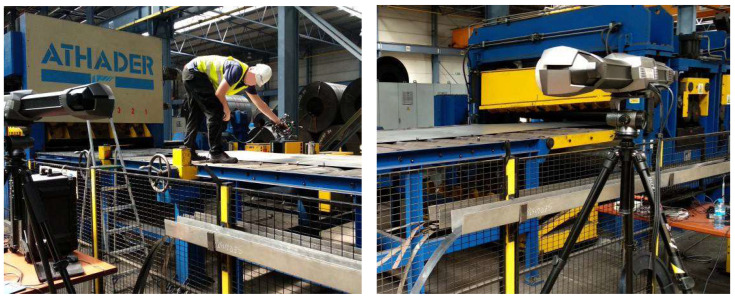
Sheet scanning using a 3D laser scanner. Own elaboration.

**Figure 5:**
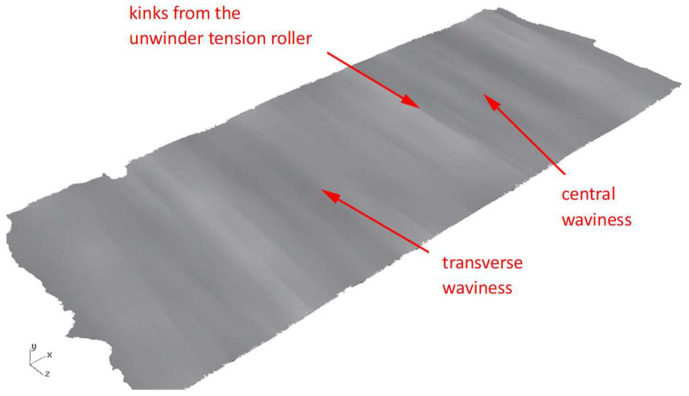
Sheet surface from the end of the coil obtained by 3D laser scanning for the steel grade S235JR + AR.

**Figure 6:**
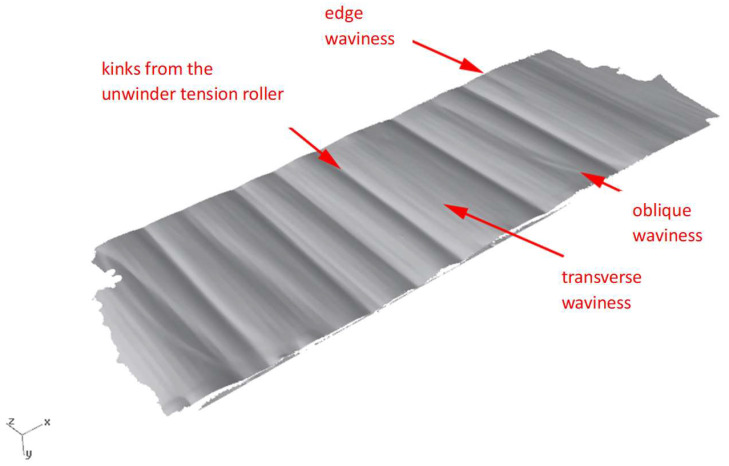
Sheet surface from the end of the coil obtained by 3D laser scanning for the steel grade S500MC.

The final stage of the preliminary research involved analyzing the geometry of the selected sheets using 3D laser scanning. These tests were carried out with a Creaform MetraSCAN 3D optical-laser scanner (AMETEK Inc. Lévis, AC, Canada), used together with the C-Track tracking system, enables precise measurement of components. In combination with the HandyPROBE (AMETEK Inc. Lévis, QC, Canada) measuring device, sheet scanning within a range of 1 to 10 m is possible. An example view of the scanning process is shown in Figure 4, while the obtained sheet surface geometries are presented in Figure 5 and Figure 6.

**Figure 7:**
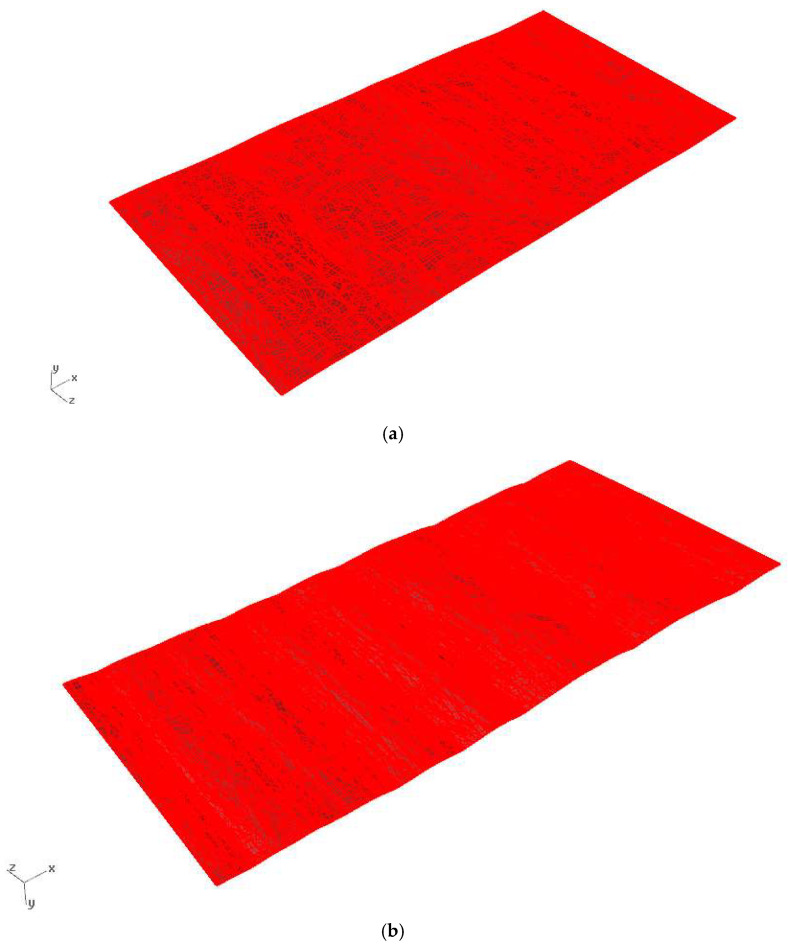
Sheet surface from the end of the coil obtained by 3D laser scanning: (**a**) for steel grade S235JR + AR and (**b**) for steel grade S500MC.

Based on the conducted scans, it was possible to determine the geometric parameters of the examined sheets and subsequently create the actual geometries to be used as input material for FEM simulations. An example view of the prepared simulation file is shown in Figure 7.

## 3. Validation of the Mathematical Model of the Material Database

The mathematical model—material database—is one of the key elements for correctly performing numerical simulations [35,36,37]. Many authors point out that the accuracy of calculations based on plasticity curves is insufficient [36,37,38], and standard models are increasingly replaced with multi-parameter curves [29,39,40,41]; however, this approach is not always adequate. Therefore, as part of the work related to modeling the reduction in internal stresses and defects in uncoiled sheet metal, it was necessary to carry out validation of the obtained material model.

**Figure 8:**
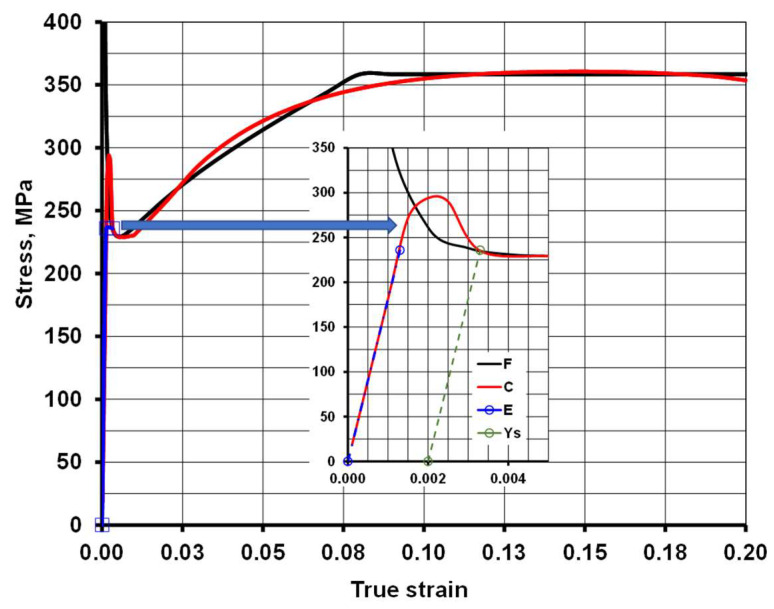
Graphical representation of the mathematical models of flow stress for the exemplary steel grade S235JR + AR (C—actual curve; E—Young’s modulus; F—four-parameter stress function; Ys—flow stress).

As part of the model verification, a comparison was made between the actual flow stress curves and those generated numerically based on Equation (1) and the coefficients listed in Table 7. Figure 8 presents an example of such a comparison for steel grade S235JR + AR. In Table 8 is presented the geometric parameters of the sheet waviness measurement for the selected steel grades using the 3D laser scanning method after uncoiling. 

**Figure 9:**
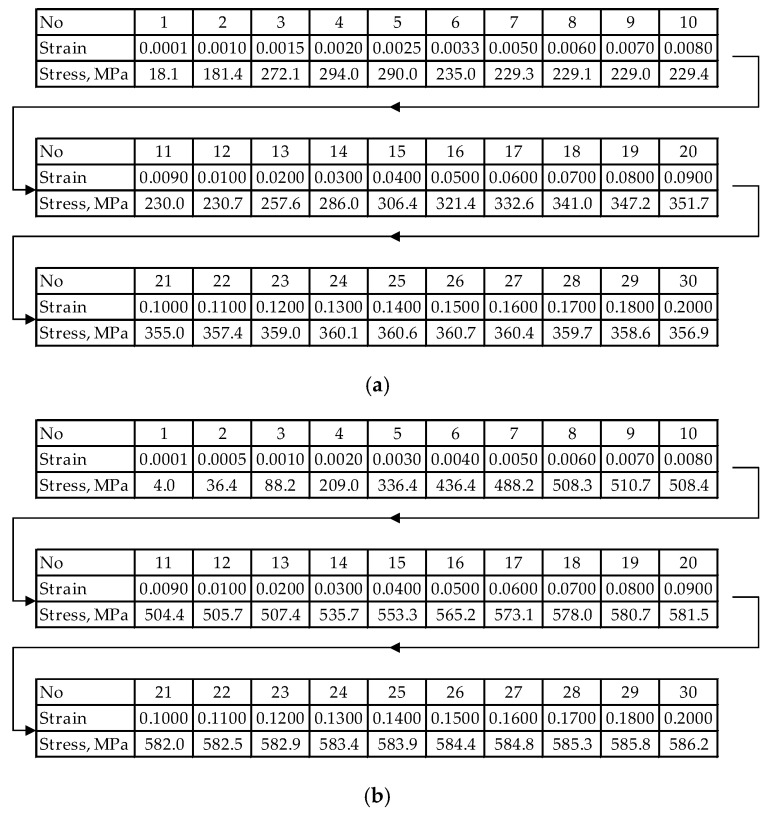
The flow stress value σ_f_ for temperatures 10–100 °C, strain rates 0.0001–100 s^−1^ and true strains in the range 0.0001–0.2 presented in tabular form: (**a**) steel S235JR + AR, (**b**) steel S500MC.

By analyzing the course of stress changes as a function of strain, it can be observed that discrepancies occur between the curve determined during experimental tests and the curve obtained by approximating the results of static tensile tests. This is particularly noticeable in the region where the material transitions into the plastic state (the area marked with an arrow and enlarged). An imprecise mathematical description of the flow stress value (σ_f_) may be one of the causes of significant numerical errors during tensile test simulations (determination of the range of plastic deformation). To reduce the discrepancies between the approximated and the actual results, it is necessary to introduce the flow stress values in a tabular form, without approximating the static tensile test results. When constructing such a table, it should be assumed that the accuracy of the flow stress values after approximation depends on the number of input data points for the actual strain and stress σ_f_ values. Reading the flow stress values for real strain increments equal to 0.05 may, in some strain ranges, result in underestimated σp values due to the adopted computational step size. Based on the obtained results of static tensile tests for individual steel grades, tabular forms of the flow stress were developed, as shown in Figure 9.

Based on the obtained data for the flow stress region, the differences between the experimental and simulation results were determined for two variants: one using the flow stress function and the other using the tabular form of the flow stress. The values of the relative percentage differences are presented in Table 9.

As can be observed, the obtained differences in stress values fall within the range of 0.63–2.35%, which indicates that the proposed testing procedure is highly accurate and can be applied to determine the range of forces and elongations. The developed mathematical model of flow stress enables computer simulation of the sheet metal using a model of the deformable object in the elastic–plastic state. The elastic state is represented by Young’s modulus, while the deformation range beyond the yield point is implemented using the actual values taken from the table. This is particularly important when testing at ambient temperature. In such cases, models using approximation curves exhibit greater error in the area of elastic and small plastic strains.

## 4. Numerical Analysis of Sheet Straightening in a Roll Straightener

**Figure 10:**
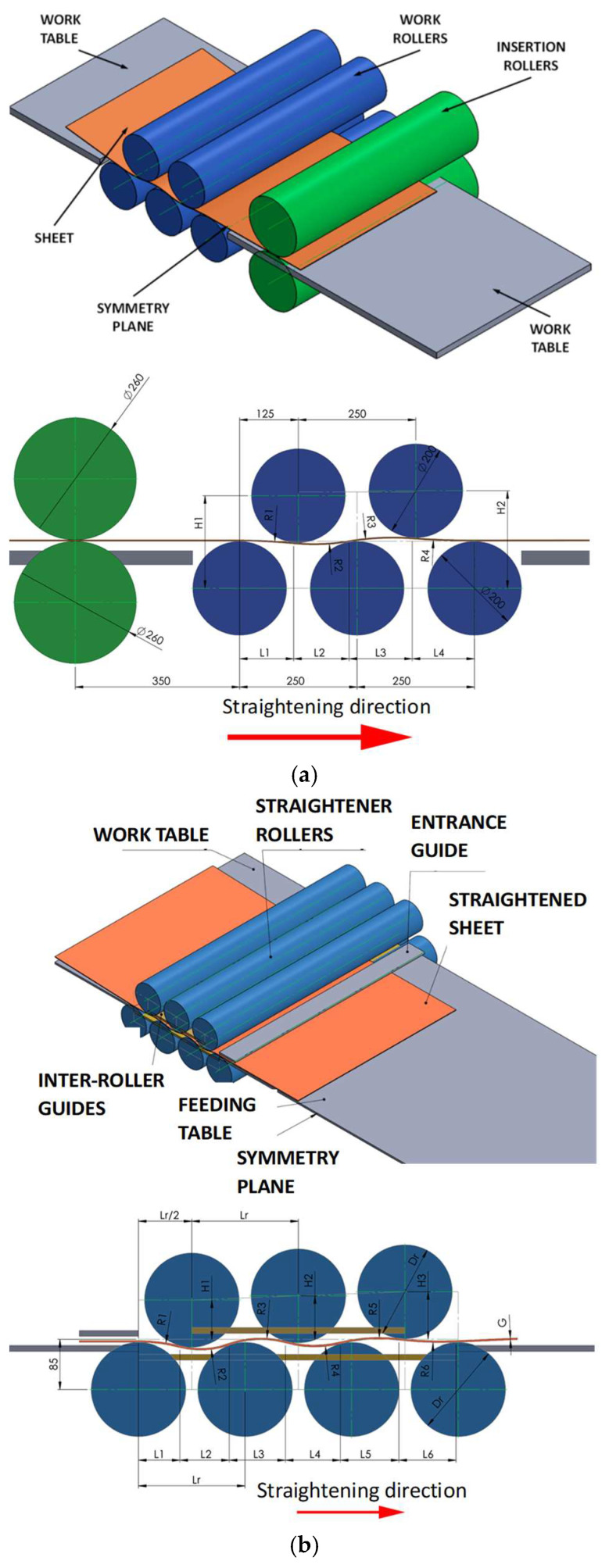

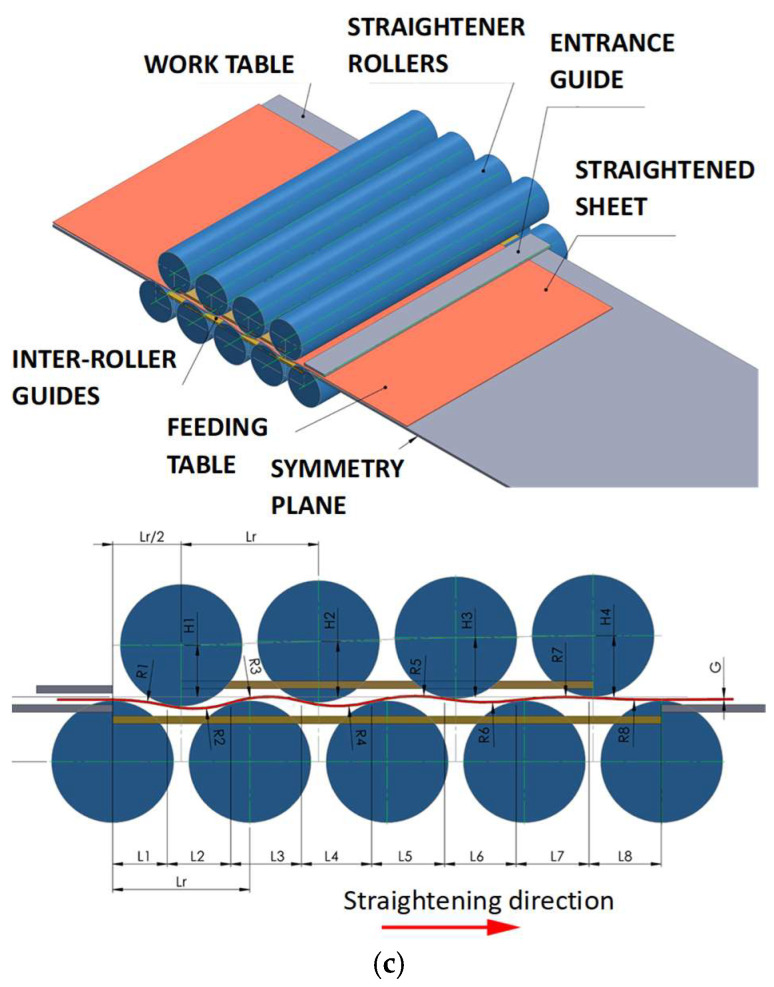
Diagram of the roll straightener and the models used for computer simulation of sheet straightening: (**a**) five-roll, (**b**) seven-roll, (**c**) nine-roll.

As part of the research continuing the work presented in [4], computer simulations of the straightening process were carried out using a roll straightener equipped with 5 rolls with a diameter of 200 mm, and 7 and 9 rolls with a diameter of 160 mm. The study was conducted using the software ForgeNxT^®^. The simulation used the rigid bodies model for the tools, this simplification was used because the aim of this research was the selection of roll pre-leveler parameters to ensure the removal of so-called “coil memory”, reducing waviness (geometry defects). The 5-roll straightener is treated as the reference model. Numerical modeling of the sheet metal rolling process is a challenging task, primarily due to geometric factors—specifically the high width-to-thickness ratio. For the calculations, an elasto-plastic model of the deformed material was applied, disregarding the influence of strain rate. Material properties were determined based on tensile tests; the resulting tensile curves were converted into stress–strain curves and implemented as constitutive data. From the perspective of sheet straightening, properties in the longitudinal direction are of paramount importance. The strip geometry, obtained through 3D scanning, was introduced as a boundary condition. Contact between the material and the rollers was defined using the Coulomb friction model. Due to the unfavorable thickness-to-width ratio, the analysis of stress–strain states was performed using localized mesh refinement. These areas were kept small to allow for a finite element mesh dense enough to ensure sufficiently accurate results. The computer simulation of the leveling process was carried out with the use of an elastoplastic model in the triaxial state of strain by using the ForgeNxT^®^ v2.1 Transvalor program, whereas the properties of the deformed material were described according to the Norton–Hoff [42,43] conservation law. The application of the computer program ForgeNxT^®^ using the thermo-mechanical models that it contains requires the definition of boundary conditions, which are decisive to the correctness of numerical computation. The theoretical analysis was performed for the following conditions: friction coefficient, *µ*—0.15 (according to the ForgeNxT^®^ v2.1 Transvalor database and [44]); sheet temperature—20 °C; Poisson coefficient—0.3, leveling velocity 45 m/min., number of elements 210,000; specific heat—480 J/(kg·K); density—7850 kg/m^3^; conductivity—29.9 W/(m·K). Figure 10 presents the diagrams of the roll straighteners and the models used for the simulations.

**Figure 11:**
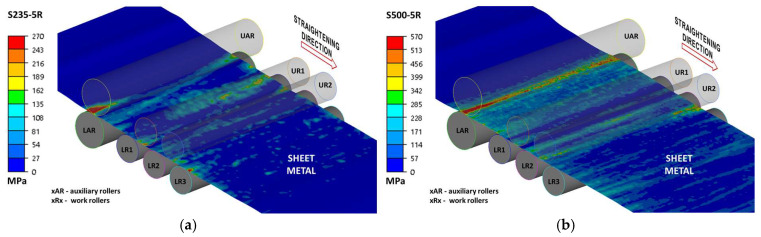
Equivalent stress distribution during sheet five-roll straightening: (**a**) steel S235 + AR, (**b**) steel S500MC.

**Figure 12:**
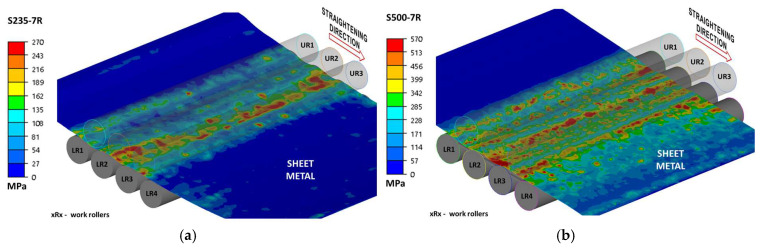
Equivalent stress distribution during sheet seven-roll straightening: (**a**) steel S235 + AR, (**b**) steel S500MC.

**Figure 13:**
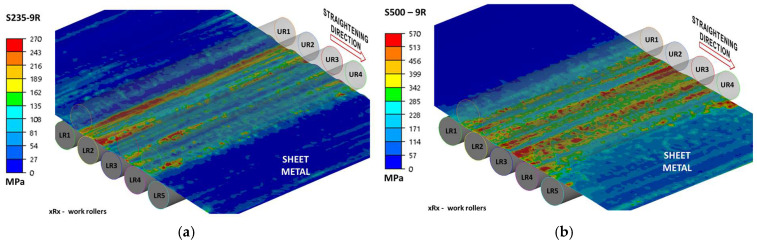
Equivalent stress distribution during sheet nine-roll straightening: (**a**) steel S235 + AR, (**b**) steel S500MC.

Due to the complexity of the results, the data are presented in two ways: as projections of stress intensity distributions (Figure 11, Figure 12 and Figure 13) and as side views, which also allow for a better assessment of the cross-sectional geometry. Due to the fact that the present work was carried out as part of a larger project and some of the results were presented, among others, in [4], the authors will not duplicate the results.

**Figure 14:**
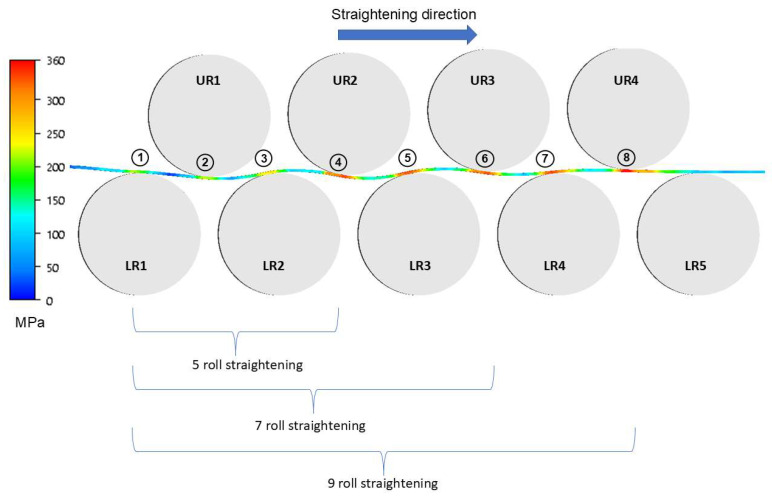
The view of the areas (measuring points) in which measurement data were collected.

In the following part of the study, the results are presented in a side view. This approach also made it possible to evaluate the geometry of the strip. Figure 14 shows a view of the areas (measuring points) in which measurement data were collected. To limit the graphical data in this article, the results from the measurement points are presented in Table 10. The mesh was selected in such a way that when the box option was used, compaction occurred in the areas of contact with the rollers. This mesh densification approach not only accelerated calculations but, more importantly, improved their quality.

Based on the data shown in Figure 14 and Table 10, it can be observed that the values of stress intensity and strain intensity, similarly to the seven-roll straightener, increase in the areas where the straightened sheet contacts the rolls, and then decrease in the free-forming regions of the sheet between consecutive rolls. However, when using the nine-roll straightener, the increase in stress and strain within the straightened sheet is much more gradual—both compared to the currently used five-roll straightener and the newly designed seven-roll straightener. Analysis of the obtained numerical simulation results showed that for the remaining components of stress and strain, the distributions are similar to those obtained for the seven-roll straightener, but the nature of their variation becomes smoother due to the addition of an extra pair of rolls. An important observation is the increase in plastic deformation through the thickness of the straightened sheet as a result of adding more straightening rolls. Therefore, increasing the number of rolls in this case is fully justified. Interesting distributions were obtained for the elastic and plastic strain intensities. While the plastic strain intensity decreases as the sheet moves between subsequent rolls—which is a natural consequence of smaller deflections of the following rolls—the elastic strain intensity increases as the sheet passes through successive rolls. This indicates that the plastic deformation in the subsequent bending zones of the sheet has an increasingly smaller effect, and thus the sheet begins to deform mainly within the elastic range. A similar relationship was noted for the total strain in the straightened sheet, which is plastically deformed. However, in the case of the nine-roll straightener, plastic deformations are distributed over the first seven rolls, where the total strain reaches its highest and most intense values (as a sum of elastic and plastic strains), and then decreases sharply in the last two rolls, where the sheet is deformed only elastically.

**Figure 15:**
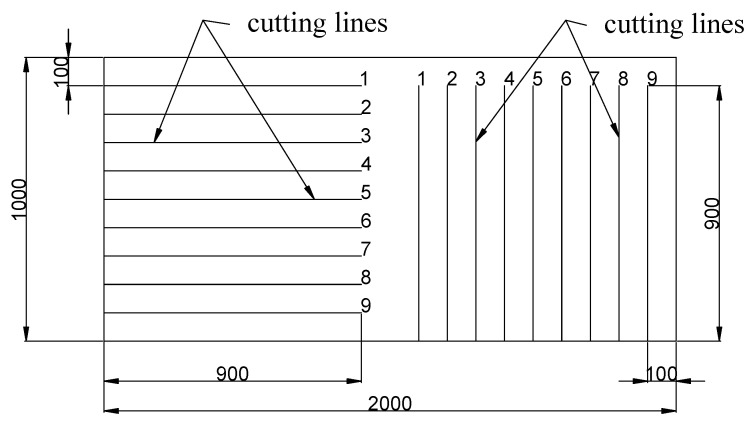
Sample preparation diagram for the author’s method of assessing straightening effectiveness.

**Figure 16:**
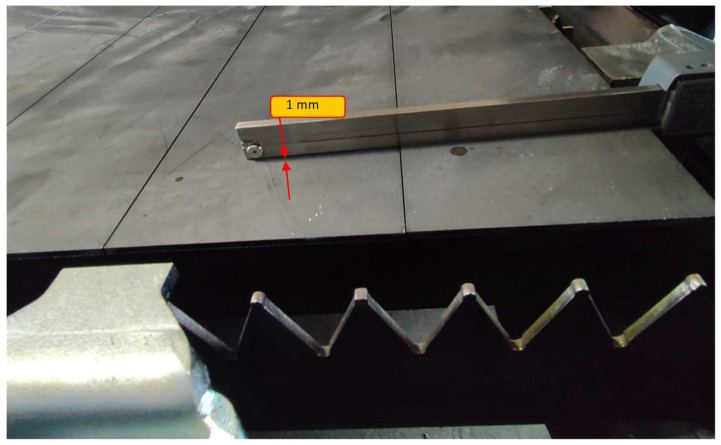
Example result of strip bending measurement for a 3 mm thick sheet of S235JR steel.

In summary, the results of the numerical calculations for the five-roll straightener and the seven- and nine-roll straighteners indicate that increasing the number of rolls positively affects the plasticization of the sheet. The plasticization process becomes smoother, and increasing the number of rolls to nine improves the straightness of the sheet and enhances the reduction in residual stresses—one of the main objectives of using a preliminary multi-roll straightener. Due to the fact that, based on numerical data, it was determined that the most effective system was one containing nine rollers for physical testing, such a system was selected. As mentioned, the research was conducted with an industrial partner, and therefore, one of the goals was to develop inspection tools suitable for such conditions. For this purpose, an original tool was developed in the form of a specially prepared laser-cut sheet of metal. A sample view is presented in Figure 15, and the results of measurements performed with commonly available engineering tools are presented in Table 11 and Figure 16.

The choice of the method for assessing the state of internal stresses was based on the assumption that a material with trapped stresses in its volume will lead to their release and change in geometry. An easy and clear comparison result is the deflection measurement shown in the data in Table 10.

## 5. Conclusions

The numerical studies carried out demonstrated a high sensitivity of the input parameters to the obtained results. The validation of the mathematical model of the material database, combined with the integration of tabular data and real data from sheet surface scans, made it possible to achieve highly accurate results. Based on the obtained research findings, the following statements and conclusions can be formulated:

1. The anisotropy of the examined sheets, determined both by hardness measurements and static tensile tests, is evident. Therefore, for proper numerical analyses, it is essential to average the real material properties based on the obtained data.
2. To perform computer simulations with higher computational accuracy, it was necessary to transition from a material model based on the flow curve equation to a tabular form of data.
3. The incorporation of real sheet geometries, correlated with actual anisotropy properties, allowed for a high degree of accuracy in the obtained research results.
4. In the case of the nine-roll straightener, plastic deformations are distributed over the first seven rolls; total deformations reach their highest and most intense values (as a sum of elastic and plastic strains), and then decrease sharply in the last two rolls, where the sheet is deformed only within the elastic range.
5. On the base of numerical simulation it can be stated that use of a nine-roll configuration has a positive effect on the sheet straightening capability.
6. The proprietary method of analyzing the state of internal stresses enables a quick and rough assessment that can be applied in industrial conditions.
7. The measurements of real materials showed some differences between the tested steel grades and confirmed that the use of a nine-roll straightening system significantly reduces the stress on the material.

## Figures and Tables

**Table 1:** Chemical composition (wt.%) of analysed materials.

	C	Si	Mn	P	S	Al	Fe
S235JR + AR	0.22	0.05	1.62	0.052	0.048	0.014	ball.
S500MC	0.11	0.45	1.55	0.021	0.010	0.012	ball.

**Table 2:** Designation of samples for hardness testing.

Steel Grade	Sample Designation	Sampling Location
S235JR + AR	1 (b,m,e)	middle
	1_1 (b,m,e)	edge
S500MC/3	2 (b,m,e)	middle
	2_1 (b,m,e)	edge

**Table 3:** Hardness measurement results.

Des.	1	2	3	4	5	Mean	Std. Dev.	% Difference
1b	127.7	128.8	128.9	127.8	128.5	128.3	0.56	
1_1b	135.5	136.7	135.2	133.4	131.4	134.4	2.07	4.75
2b	229.8	226.4	226.3	229.4	227.1	227.8	1.68	
2_1b	232.9	235.7	234.5	237.6	233.9	234.9	1.81	3.13
1m	126.3	127.2	127.9	128.5	129.7	127.9	1.29	
1_1m	132.1	131.0	132.4	134.2	132.1	132.4	1.16	3.47
2m	228.9	225.3	226.5	224.7	225.1	226.1	1.70	
2_1m	229.6	230.1	231.2	234.6	237.9	232.7	3.51	2.91
1e	128.7	127.8	128.9	128.5	129.7	128.7	0.69	
1_1e	142.1	144.2	142.8	138.0	139.0	141.2	2.62	9.71
2e	231.8	234.4	233.4	236.1	234.1	234.0	1.56	
2_1e	239.9	238.7	237.9	238.5	239.4	238.9	0.78	2.10

**Table 4:** Anisotropy of the sheet made of S235JR + AR Steel.

					Average Across Width	Difference, %
		Beginning of coil				
		Edge 1	Center	Edge 2		
	Ys, MPa	238	227	248	237.7	8.5
	UTS, MPa	364	353	375	364	5.9
		Middle of coil				
		Edge 1	Center	Edge 2		
	Ys, MPa	231	226	234	230.3	3.4
	UTS, MPa	353	351	352	352.0	0.3
		End of coil				
		Edge 1	Center	Edge 2		
	Ys, MPa	237	230	244	237.0	5.7
	UTS, MPa	365	346	381	364.0	9.2
Average along coil length	Ys, MPa	235	228	242	235.0	5.9
	UTS, MPa	361	350	369	360.0	5.1
	Ys, %	0.3	2.0	1.6		
	UTS, %	0.3	2.0	6.1		

**Table 5:** Anisotropy of the sheet made of S500MC steel.

					Average Across Width	Difference, %
		Beginning of coil				
		Edge 1	Center	Edge 2		
	Ys, MPa	364	353	375	364.0	5.9
	UTS, MPa	520	499	535	518.0	6.7
		Middle of coil				
		Edge 1	Center	Edge 2		
	Ys, MPa	353	351	352	352.0	0.3
	UTS, MPa	510	500	506	505.3	1.2
		End of coil				
		Edge 1	Center	Edge 2		
	Ys, MPa	365	346	381	364.0	9.2
	UTS, MPa	506	489	516	503.7	5.2
Average along coil length	Ys, MPa	360.7	350.0	369.3	360.0	5.1
	UTS, MPa	512.0	496.0	519.0	509.0	4.4
	Ys, %	0.3	2.0	1.6		
	UTS, %	3.0	2.0	6.1		

**Table 6:** Characteristics of averaged yield stress curves.

Steel Grade	Conventional Yield Strength Ys, MPa	Tensile Strength UTS, MPa	Young’s Modulus E, GPa	Maximum Absolute Error Δ, MPa	Maximum Relative Error δ, %
S235JR + AR	234	360	181	±8.25	2.29
S500MC	509	582	169	±10.63	1.83

**Table 7:** Coefficients of Function (1) Defining the Flow Stress.

Steel Grade	K	m_1_	m_2_	m_3_
S235JR + AR	483	0.18	0.001	1.8
S500MC	787	0.12	0.001	0.01

**Table 8:** Geometric parameters of the sheet waviness measurement for the selected steel grades using the 3D laser scanning method after uncoiling.

Steel Grade	Sheet Thickness, mm	Sheet Width, mm	Average Waviness Height, mm		Waviness Wavelength, mm
			Center of the Coil	End of the Coil	
S235JR + AR	3	1500	15.2	21.5	Irregular
S500MC	3	1500	29.7	34.8	Irregular

**Table 9:** The values of the relative percentage differences.

Steel Grade	Tensile Force F0.2 kN Experiment	Tensile Force F0.2 kN FEM Table	Tensile Force F0.2 kN FEM Function	Difference Experiment/FEM Table %	Difference Experiment/FEM Function %
S235JR + AR	13.964	13.903	12.389	−0.4	−4.2
S500MC	30.170	29.866	26.238	−1.0	−2.7

**Table 10:** The results of stress and strain from the measurement points.

S235	Straightening	Roll								Sum	Upper Rolls Settings			
		1	2	3	4	5	6	7	8		UR1	UP2	UP3	UP4
Strain	5	0.0029	0.0066	0.0057	0.0042	x	x	x	x	0.0193	12.5	1.0	x	x
Stress	5	231.70	252.97	248.99	241.25	x	x	x	x					
Strain	7	0.0025	0.0063	0.0073	0.0074	0.0065	0.0056	x	x	0.0356	6.0	3.5	1.0	x
Stress	7	227.73	251.71	255.99	256.17	252.83	248.68	x	x					
Strain	9	0.0016	0.0043	0.0057	0.0062	0.0062	0.0060	0.0055	0.0050	0.0403	4.0	3.0	2.0	1.0
Stress	9	217.29	241.66	248.99	251.54	251.33	250.31	248.02	245.45					
S500MC	Straightening	Roll								Sum	Upper Rolls Settings			
		1	2	3	4	5	6	7	8		UR1	UP2	UP3	UP4
Strain	5	0.0033	0.0071	0.0063	0.0052	x	x	x	x	0.0219	13.0	2.5	x	x
Stress	5	501.06	517.00	514.57	510.42	x	x	x	x					
Strain	7	0.0027	0.0067	0.0077	0.0075	0.0071	0.0067	x	x	0.0385	6.5	3.5	2.5	x
Stress	7	454.44	515.82	518.44	518.13	516.97	515.82	x	x					
Strain	9	0.0020	0.0053	0.0068	0.0074	0.0072	0.0068	0.0064	0.0061	0.0479	5.0	4.0	2.5	2.0
Stress	9	340.76	510.85	516.08	517.76	517.04	515.86	514.63	513.61					

**Table 11:** Example measurement results for the tested materials.

Grade	Measurement Direction	Deflection Value, mm										Mean
		1	2	3	4	5	6	7	8	9	10	
S235JR	Along the straightening	1.82	1.15	0.95	1.23	1.06	0.86	1.32	1.26	1.46	1.48	1.26
	Across straightening	0.78	0.71	0.63	0.51	0.49	0.49	0.41	0.53	0.54	0.76	0.59
S500MC	Along the straightening	1.55	1.89	1.21	1.47	1.51	1.41	1.74	1.41	1.63	1.27	1.51
	Across straightening	0.99	0.84	0.72	0.68	0.79	0.57	0.71	0.73	0.81	0.61	0.75

## Data Availability

The raw data supporting the conclusions of this article will be made available by the authors on request.
